# Detection of porcine circovirus genotypes 2a and 2b in aborted foetuses from infected swine herds in the State of São Paulo, Brazil

**DOI:** 10.1186/1751-0147-54-29

**Published:** 2012-05-03

**Authors:** Alessandra MMG de Castro, Taís F Cruz, Vanessa R Salgado, Tatiana M Kanashiro, Karen L Ferrari, João P Araujo, Paulo E Brandão, Leonardo J Richtzenhain

**Affiliations:** 1 Department of Preventive Veterinary Medicine and Animal Health, College of Veterinary Medicine, University of São Paulo (USP), São Paulo, SP, 05508-270, Brazil; 2 Department of Immunology and Microbiology, Institute of Biosciences, College of Veterinary Medicine, São Paulo State University (UNESP), Botucatu, SP, 18618-000, Brazil

**Keywords:** Pig, Abortion, Porcine circovirus, Porcine parvovirus, *Brucella*

## Abstract

**Background:**

Porcine circovirus type 2 (PCV2) has been associated with several disease complexes, including reproductive failure. The aim of this study was to identify the subtypes of PCV2 that are associated with reproductive failure in pigs from the State of São Paulo, Brazil and to investigate co-infections with other infectious organisms.

**Findings:**

Samples of 168 aborted foetuses or mummified foetuses from five farrow-to-finish swine farms known to be infected with PCV2 and located in the State of São Paulo were tested for PCV2 by polymerase chain reaction (PCR). Positive samples were additionally tested for porcine parvovirus (PPV), *Leptospira* spp. and *Brucella* spp. by PCR. PCV2 was detected in 18 of the samples (10.7%). PPV, *Brucella* spp. and *Leptospira* spp were found in 2, 10 and 0 cases, respectively. Eleven PCV2 strains were sequenced and determined to be either genotype 2a (n = 1) or 2b (n = 10).

**Conclusions:**

The findings indicate that the frequency of PCV2 infections in aborted porcine foetuses from the State of São Paulo is rather low (10.7%) and that co-infection with other pathogens is common and may be involved in PCV2 associated reproductive failure. No repeatable, characteristic amino acid motifs for regions of the PCV2 capsid protein seemed to be associated with abortion in sows.

## Findings

Porcine circovirus type 2 (PCV2) is a member of the family *Circoviridae* and the genus *Circovirus*. PCV2 is further grouped into genotypes a, b and c. PCV2a and PCV2b occur worldwide, while PCV2c was recently detected in archival samples in Denmark [1]. The virulence of the subtypes has not yet been determined. A predominance of type 2b in herds having PCV2 associated diseases (PCVAD) has been described in several countries in Europe and in North America, while PCV2a has been recovered before PCVAD were recorded in Canada, in non-PCVAD affected farms in the USA and in healthy animals in Switzerland [2,3]. In Brazil, both PCV2 genotypes a and b have been detected but a correlation between genotype and disease has not been observed [4].

An association between PCV2 infection and reproductive failure has been reported both in field and experimental studies [5]. Given the distribution of the virus in swine populations worldwide and the impact of PCV2 associated reproductive failure on swine herd economy, the aim of this study was to identify genotypes of PCV2 involved in reproductive failure in State of São Paulo, Brazil and to investigate the level of co-infections of foetuses with other pathogens.

Samples were obtained from 168 incidents of reproductive failure occurring between 2007 and 2009 in five known PCV2 infected farrow-to-finish swine farms with a history of PCVAD and located in the State of São Paulo. One foetus was examined from each incident and consisted of a mummified (n = 44) or an aborted foetus (n = 124).

PCVAD was defined as a significant increase in the post-weaning mortality and ‘wasting’ rates and detection of PCV2 nucleic acid by polymerase chain reaction (PCR) in 50% of pigs that died with clinical signs of PCVAD in the post-weaning period.

Sampled tissues were pooled for PCR testing and consisted of spleen, liver, lung, heart and kidney from aborted foetuses and the abdominal organs from mummified foetuses. Samples were homogenized using a Stomacher 80 (Seward/Lab System) in 20% (v/w) TE buffer (10 mM Tris-HCl, 1 mM EDTA, pH 8.0) and stored at -80°C until DNA extraction was carried out according to the procedure described by Chomkzynski [6]. Tissue pools were analysed for PCV2 by PCR using the primer pair Fa/Ra, which amplifies a 476 bp fragment [4]. PCV2 positive samples were additionally tested for *Brucella* spp, *Leptospira* spp. and. porcine parvovirus (PPV) by PCR. *Brucella* spp. DNA was detected using the genus specific primer pair ITS66/ITS279 [7]. The PCR assay for the detection of *Leptospira* spp. was conducted using the primer pair Lep1/Lep2, which amplifies a 331 bp fragment from the 16S rRNA gene of *Leptospira* spp [8]. Finally, PPV DNA amplification was achieved using a pair of primers directed to the VP2 sense/antisense template that amplifies a 197 bp fragment [9]. To avoid false-negative PCR results due to failures during DNA isolation or the presence of PCR inhibitors, the extracted DNA was tested in parallel for the amplification of β-actin as a PCR control [10]. Positive controls (PCV2, PPV, *Leptospira* spp. and *Brucella* spp. templates) and negative controls (sterilised MilliQ water) were used in each reaction. McNemar’s test for the comparison of paired proportions was used to determine the significance of differences (*P* < 0.05) between PCV2 positive samples and presence of the other organisms.

To determine the extent of genetic diversity, the capsid gene was sequenced using two pairs of primers (P5/P6 and P7/P8) [11]. Bi-directional sequencing reactions were performed using BigDye^TM^ Terminator v3.1 (Applied Biosystems™) and run using an ABI PRISM® 377 DNA Sequencer (Applied Biosystems). The consensus sequence assembly was performed using the PHRED/PHRAP and CAP3 (http://bioinformatica.ucb.br/electro.html) programs with an analysis quality point of 20. The obtained sequences were aligned with sequences from GenBank using Clustal X software [12]. Phylogenetic analysis was performed on the aligned data set and a rooted tree was constructed in MEGA v2.1 using the distance-based neighbor-joining method with the PCV type 1 sequence as an out-group (GenBank number AY184287) and the PCV2 sequences available from GenBank for genealogical analysis. Bootstrap values were calculated on 1000 repeats. The rooted tree was created using Tree Explorer from the MEGA v2 package.

PCV2 was detected in 10.7% (18/168) of the samples and as a mono-infection in 3.57% (6/168). Of the 18 PCV2 positive cases, PPV, *Brucella* spp., and *Leptospira* spp. were identified in 2, 10 and 0 cases, respectively. Dual or triple infections including PCV2 consisted of PCV2a and PPV (n = 1), PCV2b and *Brucella* spp (n = 10), and PCV2b and PPV (n = 1). A significant difference was observed between the prevalence of dual or triple infections (PCV2 and others agents) and PCV2 mono-infections using the McNemar’s test (*P = 0.031*).

Fragments of 130 nt and 196 nt from three *Brucella* positive cases and two PPV positive cases were sequenced and compared with sequences available from the GenBank database demonstrating a nucleotide identity of ≥95% (data not shown).

**Figure 1:**
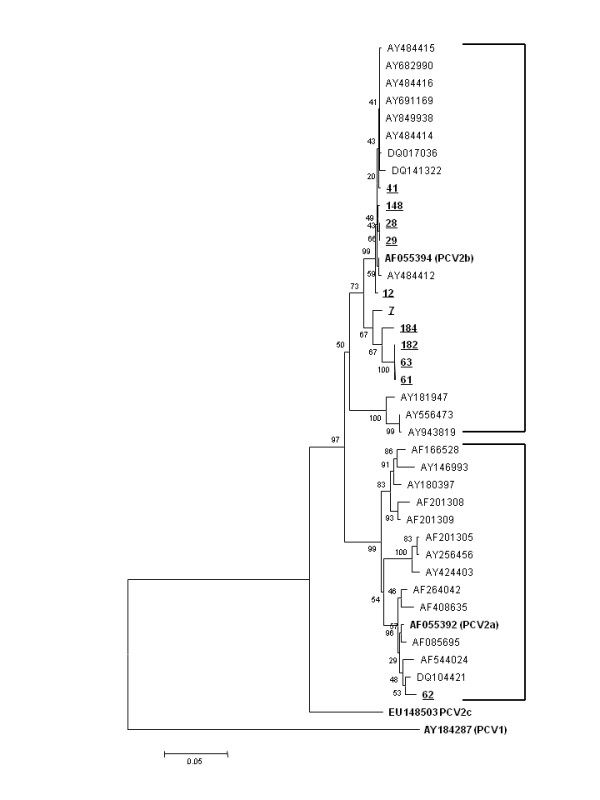
**Phylogenetic tree based on the neighbour-joining method for the 40 sequences (11 from this study) used for the analyses in the study.** The numbers along the brackets refer to the percentages of confidence in the analyses. Strains are indicated by their GenBank accession numbers and the genotype reference sequence identifications are indicated in parentheses.

Eleven ORF2 sequences among the 18 PCV2 positive samples achieved proper sequence quality and were further analysed. The neighbour-joining tree that was assembled classified 10 sequences as genotype 2b and one sequence as 2a (Figure 1). The capsid protein of PCV2 was chosen because it is exposed to selective immune pressures and represents a potential candidate region involved in the emergence of PCV2 variants. However, no repeatable characteristic amino acid motifs in the PCV2 capsid protein or in the residues used for genotype classification were associated with clinical disease. The results are consistent with previous field and experimental reports, in which both genotypes have been detected and were able to induce similar gross lesions and virus replication rates as those described in PCV2 associated reproductive disorders [13,14].

The involvement of PCV2 in reproductive failure is of considerable interest but reproductive failure is not a consistent finding in outbreaks of PCVAD. Most field reports are based on accidental findings by diagnostic laboratories. Although the samples studied here were collected in PCV2 positive swine herds that had other clinical signs of PCVAD than reproductive failure, PCV2 was detected as a mono-infection in only 3.57% of the samples. The prevalence of dual or triple infections including PCV2 was higher (7.2%). Previous studies performed in Brazil have failed to detect PCV2 in samples from incidents of reproductive failures or only detected PCV2 at similar low levels (5.74%) [14-16]. The low rate of PCV2 detection in the cases studied here is similar to rates found in other studies [17]. However, the importance of the virus in some incidents of reproductive failure has been demonstrated by Lyoo *et al*. [18], who detected mono-infection with PCV2 in 4/16 aborted foetuses in Korea. The role for PCV2 in reproductive failure was also reported from the Czech Republic, where the frequency of PCV2 isolation in association with reproductive failure increased from 2005 to 2007 [19]. The discrepancies between studies may be associated with the PCV2 load in the herds and the time of infection of gilts as demonstrated by Hansen *et al*[14]. Occurrence of PCV2 associated reproductive disorders is more common in gilts or in new herds that have recently been exposed to the virus [5].

The ability of PCV2 to infect porcine foetuses is well documented but abortion may depend on the presence of other incidents, e.g. co-infections with another virus or bacteria as found in this study. A number of studies have reported co-infections with PCV2 and PPV, and PPV has been established as an important virus involved in PCV2 associated reproductive failure [17,18]. Co-infections between PCV2 and *Brucella* spp. has not been reported previously. Despite a national control program for brucellosis in Brazil, the organism was detected in 10/18 PCV2 positive samples. Because of the importance of *Brucella* spp. as a zoonotic agent, farmers with herds that were positive for brucellosis were notified of their status to improve brucellosis control.

## Conclusion

The findings indicate that PCV2 has a limited impact on reproduction in swine herds where the sows have been exposed to PCV2 previously and where co-infections are common. The detection of brucellosis as a co-infection demonstrates that uncommon agents may be involved in PCVAD. No repeatable, characteristic amino acid motifs for regions of the PCV2 capsid protein for either genotypes a or b were associated with reproductive failure.

## Competing interests

None of the authors of this paper have a financial or personal relationship with other people or organizations that could inappropriately influence or bias the content of the paper.

## Authors’ contributions

AMMGC initiated and designed the study together with LRJ. AMMGC wrote the manuscript, interpreted the results and drew conclusions in discussion with other authors. KLF contacted the swine producers and provided the samples used in this study. AMMGC and KLF set up the PCV2 PCR and sequencing. LRJ critically revised the manuscript and gave the final approval for publication. TFC and TMK set up the PPV PCR and sequencing and helped with the writing of that part. VRS set up the *Brucella* spp PCR and sequencing and helped with the writing of that part. JPAJr drew the PPV PCR and sequencing and helped with the results interpretation of that part. PEB helped with the sequencing PCV2 analyses and helped with the results interpretation of that part. All authors read and approved the final manuscript.
